# Post‐task responses following working memory and movement are driven by transient spectral bursts with similar characteristics

**DOI:** 10.1002/hbm.26700

**Published:** 2024-05-10

**Authors:** Sebastian C. Coleman, Zelekha A. Seedat, Daisie O. Pakenham, Andrew J. Quinn, Matthew J. Brookes, Mark W. Woolrich, Karen J. Mullinger

**Affiliations:** ^1^ Sir Peter Mansfield Imaging Centre, School of Physics and Astronomy University of Nottingham Nottingham UK; ^2^ Young Epilepsy Lingfield UK; ^3^ Clinical Neurophysiology Queen's Medical Centre, Nottingham University Hospitals NHS Trust Nottingham UK; ^4^ Oxford Centre for Human Brain Activity, Wellcome Centre for Integrative Neuroimaging, Department of Psychiatry University of Oxford Oxford UK; ^5^ Centre for Human Brain Health, School of Psychology University of Birmingham Birmingham UK

**Keywords:** bursting, hidden Markov model, magnetoencephalography, neural oscillations, post‐movement beta rebound, post‐stimulus, rebound

## Abstract

The post‐movement beta rebound has been studied extensively using magnetoencephalography (MEG) and is reliably modulated by various task parameters as well as illness. Our recent study showed that rebounds, which we generalise as “post‐task responses” (PTRs), are a ubiquitous phenomenon in the brain, occurring across the cortex in theta, alpha, and beta bands. Currently, it is unknown whether PTRs following working memory are driven by transient bursts, which are moments of short‐lived high amplitude activity, similar to those that drive the post‐movement beta rebound. Here, we use three‐state univariate hidden Markov models (HMMs), which can identify bursts without a priori knowledge of frequency content or response timings, to compare bursts that drive PTRs in working memory and visuomotor MEG datasets. Our results show that PTRs across working memory and visuomotor tasks are driven by pan‐spectral transient bursts. These bursts have very similar spectral content variation over the cortex, correlating strongly between the two tasks in the alpha (*R* ^2^ = .89) and beta (*R* ^2^ = .53) bands. Bursts also have similar variation in duration over the cortex (e.g., long duration bursts occur in the motor cortex for both tasks), strongly correlating over cortical regions between tasks (*R* ^2^ = .56), with a mean over all regions of around 300 ms in both datasets. Finally, we demonstrate the ability of HMMs to isolate signals of interest in MEG data, such that the HMM probability timecourse correlates more strongly with reaction times than frequency filtered power envelopes from the same brain regions. Overall, we show that induced PTRs across different tasks are driven by bursts with similar characteristics, which can be identified using HMMs. Given the similarity between bursts across tasks, we suggest that PTRs across the cortex may be driven by a common underlying neural phenomenon.

## Graphical abstract

We investigated post‐task magnetoencephalography responses, that is, oscillatory rebounds, following working memory and movement tasks by performing a burst analysis using hidden Markov models. Results showed that these responses were driven by transient bursts whose intrinsic characteristics were remarkably similar across tasks, despite large differences in the two tasks performed.

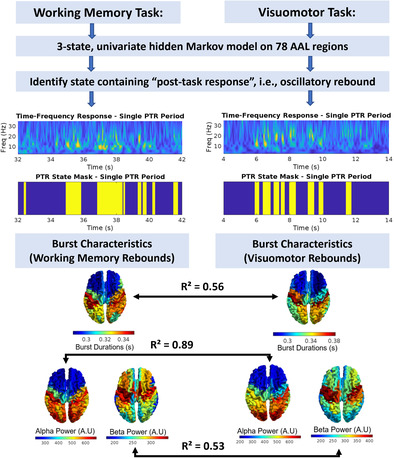

**Practitioner Points**

1. Post‐task responses in oscillatory activity following working memory and movement are explained by transient pan‐spectral bursting.
2. Burst events are captured in a single hidden Markov model state per brain region, which can be grouped across the cortex based on temporal similarity.
3. Bursts that constitute post‐task responses have similar intrinsic properties, despite being induced by different types of task.

## 1 INTRODUCTION

Post‐task responses (PTRs) are induced oscillatory responses in electrophysiological activity that occur between periods of task and rest (Mullinger et al., 2017; Pfurtscheller et al., 2005; van Zijl et al., 2012). PTRs are often referred to as rebounds when studied in the motor and visual cortices, in beta and alpha frequency bands, in response to sensorimotor and visual stimuli (Fry et al., 2016; Jurkiewicz et al., 2006; Mullinger et al., 2017; Pakenham et al., 2020; Pfurtscheller et al., 2005; Robson et al., 2016; Stevenson et al., 2011). Current evidence suggests that rebounds (periods of high amplitude alpha/beta activity) serve an inhibitory function, bringing networks that were involved in stimulus/task processing back to resting levels of activity (Chen et al., 1998; Coleman et al., 2023; Mullinger et al., 2017; Pakenham et al., 2020; Pfurtscheller et al., 2005; Solis‐Escalante et al., 2012). Our recent work has revealed that PTRs are a ubiquitous phenomenon, appearing in multiple frequency bands and locations in the brain, including higher order areas involved in working memory following an n‐back task (Coleman et al., 2023). However, it is unknown whether PTRs following working memory are driven by the same underlying neural phenomenon as those that follow movement or visual stimuli. This study uses data‐driven techniques to perform an in‐depth analysis of PTR characteristics in the human brain in response to visuomotor (Pakenham et al., 2020) and working memory (Coleman et al., 2023) tasks, with the aim of determining whether PTRs in each case are driven by similar bursting behaviour.

The most well‐characterised PTR is the post‐movement beta rebound (PMBR), an increase in beta‐band (13–30 Hz) power that is measured in the motor cortex following cessation of motor activity (Jurkiewicz et al., 2006; Pfurtscheller, 1981; Pfurtscheller et al., 2005; Spitzer & Haegens, 2017). The PMBR follows a decrease in beta‐band power that occurs during movement, termed the movement‐related beta decrease (MRBD). The PMBR has garnered interest in recent years due to its relationship with neurological disorders such as schizophrenia and autism (Gaetz et al., 2020; Gascoyne et al., 2021; Hunt et al., 2019). It has also been shown to modulate with various task parameters such as grip duration and force output (Fry et al., 2016; Pakenham et al., 2020). There is much evidence that suggests that both alpha and beta band cortical oscillations correspond to active inhibition of brain activity (Bonnefond & Jensen, 2012; Chen et al., 1998; Engel & Fries, 2010; Gaetz & Cheyne, 2006; Jensen & Mazaheri, 2010; Klimesch et al., 2007; Waldhauser et al., 2012). It follows that alpha/beta PTRs (periods of high amplitude activity) should relate to inhibitory control of networks that were active during a stimulus/task, facilitating a return to resting levels of activity. This is supported by studies showing that beta rebounds are greater in more demanding task conditions (Fry et al., 2016, Pakenham et al., 2020). Interestingly, the MRBD is invariant in cases where the PMBR is modulated (Fry et al., 2016; Gascoyne et al., 2021; Hunt et al., 2019; Pakenham et al., 2020). In addition, alpha PTRs following working memory have been shown to correlate with reaction times (RTs), a behavioural measure of task difficulty, while the responses measured during the task did not (Coleman et al., 2023). It is therefore hypothesised that PTRs may provide a unique insight into inhibitory neuronal processing which cannot be gained from responses occurring during a task (Mullinger et al., 2013; Mullinger et al., 2017; Pakenham et al., 2020).

Despite being observed directly more than 40 years ago (Pfurtscheller, 1981), the bursting nature of induced beta band activity in the motor cortex has only recently become recognised (Jones, 2016; Seedat et al., 2020; Sherman et al., 2016). Bursts are defined as short periods of high amplitude oscillatory activity, where changes in trial‐averaged power are attributed to changes in the likelihood of these events. Hidden Markov Models (HMMs) are becoming a popular tool for identifying and characterising bursting behaviour due to the data‐driven nature of state inference (Baker et al., 2014; Coquelet et al., 2022; Higgins et al., 2021; Quinn et al., 2018; Seedat et al., 2020; Vidaurre et al., 2016). Studies that have used HMMs to identify a burst state have shown that the MRBD and PMBR can be explained by changes in probability of burst events, while changes in connectivity can be explained by changes in burst coincidence between regions (Seedat et al., 2020; Vidaurre et al., 2018). This type of analysis has focussed on beta band activity, primarily in the motor cortex. It is unknown whether PTRs across the cortex following higher cognition are driven by bursts with similar characteristics to those that follow movement.

We hypothesise that: (1) PTRs are always driven by transient burst events regardless of task, (2) PTRs will have similar burst properties (spectral and duration) across different paradigms if they are driven the same neuronal processes, and (3) HMMs will isolate bursts from other brain activity and noise, strengthening previously reported relationships between PTRs and behaviour (Coleman et al., 2023). To address these hypotheses, we employ a mass‐univariate HMM approach to identify PTR states across the brain, independent of frequency band, across visuomotor and working memory task datasets. We group univariate HMM states related to PTRs using k‐means clustering, based on temporal similarity over the entire data period, allowing us to group PTR activity that occurs in different frequency bands across the cortex. We first verify that the PTR states correspond with visible bursting activity in single trial magnetoencephalography (MEG) data. We then look at spectral content and burst duration, to perform a region‐wise comparison of bursts that constitute PTRs across the two tasks. Finally, we explore the utility of HMMs for isolating signals of interest from other neuronal signals and noise by testing whether the HMM PTR state better relates to behaviour of participants than frequency filtered power envelopes (Coleman et al., 2023).

## 2 METHODS

### 2.1 Paradigms

Two datasets were analysed which had both been shown to contain PTRs which modulate with task parameters. The first was an n‐back task that incorporates elements of working memory, where PTRs modulated with working memory load (greater PTR amplitude with greater working memory load) (Coleman et al., 2023), and the second was a grip‐force visuomotor task, where PTRs modulated with grip duration (greater PTR amplitude with shorter grip duration) (Pakenham et al., 2020).

**FIGURE 1:**
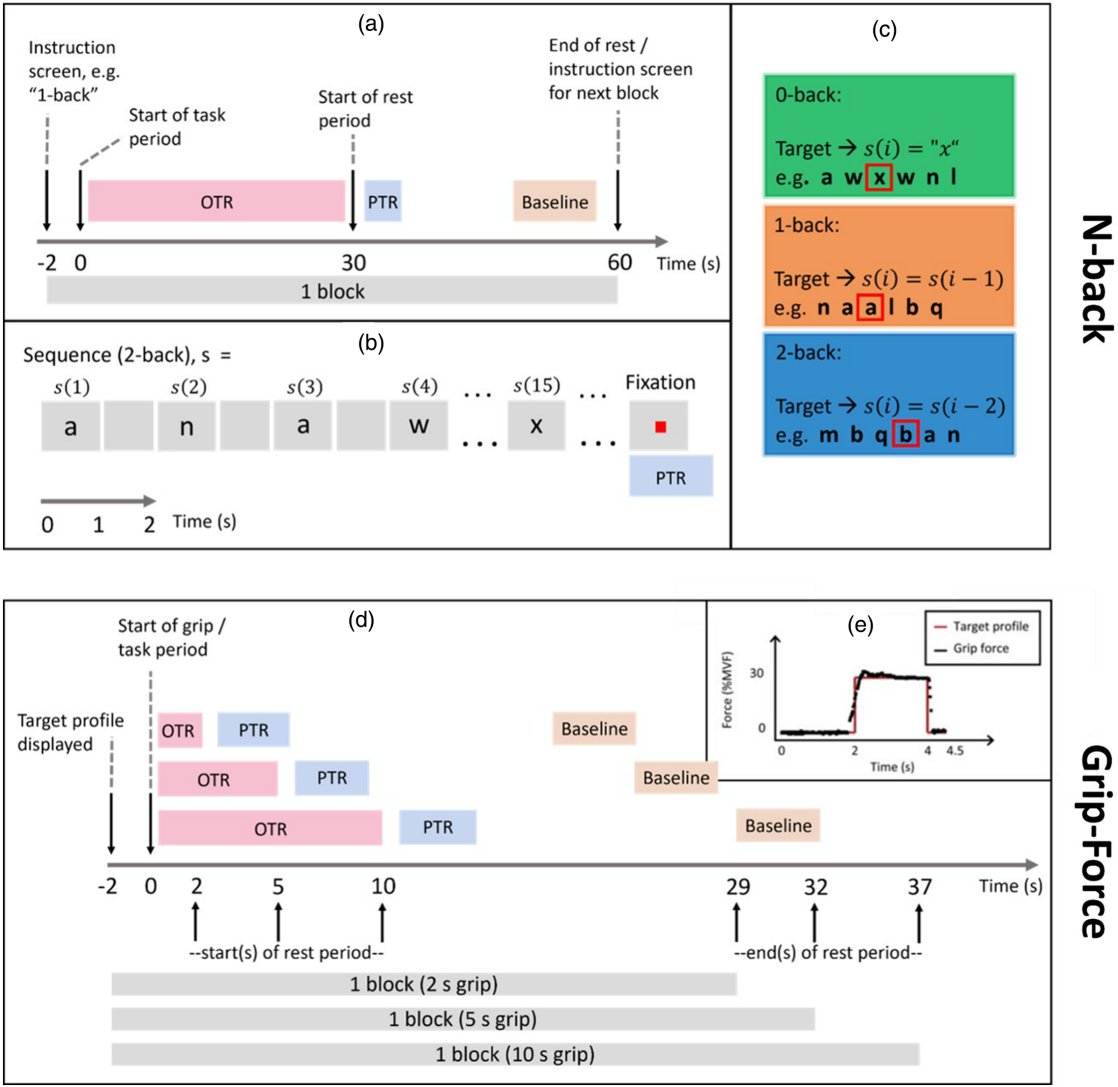
A schematic summary of the paradigms employed for the two datasets used in this study. The top panel (a–c) shows the n‐back task with 0‐back, 1‐back, and 2‐back conditions. Panel (d) shows the grip‐force task timings, with 2, 5, and 10 s grip durations, while panel (e) shows an example of the target profile and visual feedback presented to the participant. Both tasks had 30 s rest periods following each task period to facilitate the study of post‐task responses (PTRs). In these figures, the oscillatory task response (OTR) is defined as the oscillatory modulations measured during stimulus/task while PTR is the oscillatory modulations measured immediately following stimulus/task cessation, defined as 0–7 s after task cessation for the n‐back task and 2–6 s after task cessation for the grip‐force task.

The n‐back task was explained in detail in Coleman et al. (2023) and is illustrated in Figure 1a–c. In brief, during a single 30 s task period of the n‐back task, participants were presented with a series of 15 letters, each for 1 s, separated by a pause of 1 s in which a blank screen was shown. Participants were instructed to press a button with their right index finger when a target letter was shown. A target letter was either the letter “x” (0‐back), the same as the previous letter (1‐back), or the same as the letter before the previous letter (2‐back). The three conditions, 0‐back, 1‐back, and 2‐back, varied in working memory load, with 2‐back being the most demanding. Each task period was preceded by an instruction screen stating the task condition, and followed by a 30 s rest period. The instruction screen, task period and rest period made up a single experimental block lasting 62 s. Participants completed 16 blocks of each task condition, separated over 2 runs, with conditions arranged in a pseudo‐random order.

The grip‐force visuomotor task was explained in detail in Pakenham et al. (2020) and is illustrated in Figure 1d,e. In brief, it required participants to maintain 30% of their maximum voluntary force for either 2, 5, or 10 s. Participants applied this force to a grip bar, attached to the right hand with a glove (to allow the hand to fully relax during rest periods without dropping the bar). Participants were presented with a visual stimulus comprising a target force profile, which appeared 2 s before task onset. During the task, actual force output was plotted over the target profile, giving the participants visual feedback on their performance. Each task period was followed by 30 s rest. During the last 2 s of rest, the target profile for the next task period was shown. The task period and rest period make up a single experimental block. Participants completed 15 blocks of each duration per run, arranged in a pseudo‐random order, for 2 runs.

### 2.2 Data collection

Then, 20 healthy volunteers (10 female, aged 26 ± 4 [mean ± SD] years) took part in the n‐back study, and 15 healthy volunteers (10 female, aged 27 ± 3 [mean ± SD] years) took part in the grip‐force study, which were both approved by the University of Nottingham Medical School Research Ethics Committee and, where relevant, in compliance with all COVID‐19 standard operating procedures. All volunteers gave written, informed consent.

All data were collected at a sampling rate of 600 Hz using a 275‐channel CTF MEG system (MISL, Coquitlam, BC, Canada) in third order gradiometer configuration. Head localisation coils were attached to the participants' nasion and preauricular points prior to recording to provide fiducial markers for head localisation. The coils were energised before the start and after the end of each recording to provide a measure of overall head movement. The participants were scanned in a seated position for the n‐back task, and supine for the grip‐force task, viewing a projector screen that displayed the stimuli. An eyetracker (EyeLink, Ottawa, Canada) was used during the n‐back task to monitor the participants to ensure they remained awake during the relatively long rest periods.

For the n‐back task, a 3D digital mesh of the head and fiducial coils was acquired using a Skanect structure sensor (Occipital, Colorado, USA). For the grip‐force task, the 3D digitisation was performed using a Polhemus system (Polhemus, Colchester, VT, USA). The digitised head surface and fiducial locations were then co‐registered with the individual participant's anatomical MRI (T_1_‐weighted MPRAGE sequence acquired on either a 3 T or 7 T MRI scanner) to allow the position of the sensors relative to the brain to be determined.

For further details on data collection for the n‐back task, refer to Coleman et al. (2023) and for the grip‐force task, refer to Pakenham et al. (2020).

### 2.3 Pre‐processing

For the n‐back task, the sensor‐level MEG data were bandpass filtered into 1–150 Hz and DC offset was removed. Data were segmented into blocks and grouped by task condition. Each 62 s block was visually inspected and any that contained SQUID resets or excessive head movement were removed. Eye‐blink and cardiac artefacts were then removed from the remaining data using ICA in Fieldtrip (https://www.fieldtriptoolbox.org/). All 20 participants remained for further analysis after the pre‐processing.

Pre‐processing for the grip‐force dataset followed a similar procedure with the additional analysis of EMG data to define accurately the onset and offset of each grip period. For details, refer to (Pakenham et al., 2020). In this task, 14 participants remained for further analysis after the pre‐processing.

On average, 46 ± 1 (mean 0‐back: 15, mean 1‐back: 15, mean 2‐back: 15) blocks per participant remained in the n‐back dataset after removal of bad/noisy trials, while 75 ± 3 (mean 2 s: 25, mean 5 s: 25, mean 10 s: 24) blocks remained in the grip‐force dataset. Both datasets were transformed into source‐space using a linearly constrained minimum variance beamformer, using the covariance of the entire participant dataset filtered into broadband (1–150 Hz). Using the beamformer weights, timecourses were extracted from the centroids of 78 parcellated regions in the automated anatomical labelling (AAL) atlas.

### 2.4 Hidden Markov modelling

HMMs (Ossadtchi et al., 2005; Quinn et al., 2018; Seedat et al., 2020; Vidaurre et al., 2016) assume that the observed data are governed by a number of mutually exclusive hidden states, such that the data at each timepoint corresponds to one of these states. Modelling can either be multivariate, using covariance over all channels/regions, or univariate, inferring a separate set of states for each channel or region. Studies have demonstrated the utility of multivariate HMMs in measuring functional connectivity based on fast (ms) fluctuations, while univariate HMMs allow for region‐wise comparison of state characteristics (Baker et al., 2014; Coquelet et al., 2022; Quinn et al., 2018; Seedat et al., 2020; Vidaurre et al., 2016). In a time‐delay embedded HMM (Seedat et al., 2020; Vidaurre et al., 2018), the states are based on the autocovariance in a time‐window of specified width. This means that each state has an associated spectral fingerprint. We chose to run a separate time‐delay embedded HMM on each brain region to allow for region‐wise comparison of PTRs while not constraining PTRs to have the same spectral profile over the whole head. The time‐window specified in this study was 230 ms to encompass activity in the canonical frequency bands (Seedat et al., 2020). For both datasets used, we inferred HMMs with three states as we expected one state to capture PTRs, one state to capture other activity that may occur during the task (e.g., evoked responses relating to stimulus processing), and a third to satisfy the continuity requirement, that is, a state that becomes active when brain activity encompassed by the other states is *not* happening.

Before HMM inference, the unfiltered source‐space data were downsampled from 600 to 100 Hz to reduce computational expense. For each brain region in turn, data were concatenated across all participants, as well as conditions, so that a single HMM could be applied per brain region, which improves the robustness of the HMM (Seedat et al., 2020) and provides a unified description of the dynamics for each brain region. Z‐scoring was applied to the concatenated data to reduce variance between participants in the sequence. For each brain region, the posterior probabilities for each state were thresholded at two‐third (Seedat et al., 2020) to produce three binary timecourses, each showing when states are “on” and “off.” Power spectral density (PSD) profiles were extracted from the model for each state.

### 2.5 State assignment

The univariate HMM was inferred in each brain region separately, meaning that the order of inferred states was not consistent between regions. K‐means clustering was used to group univariate HMM states across regions based on temporal similarity over the entire scan. The k‐means clustering technique partitions a matrix of 234 binary state timecourses ([78 regions × 3 states] × number of timepoints) into *k* clusters by minimising the sum of squared Euclidian distances between state timecourses within each cluster. The clusters are formed in a space where each timepoint has an axis, such that a timecourse is described by a single point with position based on the value at each timepoint. Therefore, states with high functional connectivity across the brain will be readily clustered. This approach was guided by findings of Seedat et al, who showed that the MEG functional connectome is driven by the burst state (Seedat et al., 2020), which should translate to the PTR state in this study. The number of clusters was chosen to be three, as this is the minimum number of clusters required to separate the three HMM states. There are several methods of optimising the number of clusters to properly reflect the dynamics of the underlying system, however, given we were only concerned with the state that captured PTR activity, it served no purpose to infer more clusters than the number of HMM states. A simulation of this process can be seen in Figure S1. From this, we see that timecourses with similar temporal dynamics are clustered effectively until the noise level gets too high (80% noise), at which point the clusters become too poorly defined to reflect the ground truth assignments.

### 2.6 Identifying the PTR state

The first stage of analysis was to identify the PTR state cluster from the other two clusters. To do this, the binary state timecourses for each state across the brain were epoched by trial/block, and averaged to get the probability evolution over a single block, giving three state probability timecourses per AAL region, each with an associated cluster assignment label. From here, the timecourses within each cluster were averaged to obtain three probability evolution timecourses that described activity over the whole brain, that is, the three cluster centroids. From these timecourses, the PTR state cluster was identified as the one that visibly contained a PTR in the post‐task time window (0–7 s after task cessation for the n‐back task, 2–6 s after task cessation for the grip‐force task).

In addition to the probability timecourses, the Euclidian distances between each state and the cluster centroids were taken to show the quality of each cluster, that is, the amount by which the cluster centroid represents the dynamics of a given state. Within each cluster, lower Euclidian distances represent regions that are more highly connected to the cluster centroid. When comparing clusters, lower overall Euclidian distances represent a better quality cluster.

### 2.7 State analysis

Several measures were taken from the PTR state cluster in each task to determine whether the state represented similar spectral bursting in each case. First, state timings were compared visually to the MEG time–frequency response (TFR) for single data segments lasting 10 s, positioned immediately post‐task (when PTRs occur). From these, we were able to visually inspect the events in the MEG data that were encompassed in the HMM PTR state.

The next measure taken from the PTR states across the brain was spectral content. A PSD distribution was obtained for each state using the multitaper method. PSDs were clustered using k‐means clustering based on PSD morphology to aid visualisation. We then took the power (area under the curve) within three canonical frequency bands: theta (4–8 Hz), alpha (8–13 Hz), and beta (13–30 Hz), for each state/region within the PTR state cluster. These power values were plotted on a brain as a colourmap to show the spectral distribution of PTR activity across the brain. We quantitatively compared the spectral content variation across regions between the two tasks using ordinary least‐squares linear regression.

Next, state lifetimes, equivalent to burst durations, were taken from states within the PTR state cluster. These were averaged over all participants per region and displayed as a colourmap over the brain to compare bursts that drive PTRs in different brain regions. They were also averaged over all regions for each participant to compare variation of average burst duration between the two tasks. Burst durations per region were then compared across tasks using linear regression, akin to the spectral content analyses. Together the spectral content and state lifetimes were used to assess whether PTRs following visuomotor and working memory tasks are driven by the same bursting phenomenon despite being induced by very different tasks.

Finally, we reconstructed the TFR from regions with prominent PTR states in each task, using a matrix multiplication between the state probability timecourses and power spectra (three reconstructed TFRs for each region, one for each state) from the corresponding region (Quinn et al., 2018). These were compared to the actual MEG TFR from the same location to directly view the contribution of each state to the total response measured using MEG.

### 2.8 Isolating signals of interest using HMMs

In principle, if our approach is clustering together the univariate HMM states that drive PTR activity, then we are isolating the brain signals of interest, separating them from the total MEG signal which contains other neuronal signals and potentially physiological or environmental signals. This means that correlations between MEG signals and behaviour should be strengthened when using HMM state timecourses as opposed to frequency filtered MEG data from the same region. We tested this by repeating correlations reported in Coleman et al. (2023) between oscillatory modulation and RTs, for the n‐back dataset. The study reported a significant correlation (*p* < .05, simple linear regression) between RT and PTR modulation in left lateral visual alpha, as well as a trend (*p* < .1, simple linear regression) in left parietal alpha. A correlation also arose between RT and frontal theta negative PTRs after linear regression removal of the oscillatory response during the task. Here, as previously, we correlated modulation in PTR state probability with RT modulations, taking the subtraction of 2‐back and 1‐back as in Coleman et al. (2023). We restrict our analysis to anatomical areas that were examined in Coleman et al. (2023)—frontal, left parietal, left lateral visual, and the dorsal attention network (DAN). The significance of the correlations between HMM PTR and behaviour were obtained using a Pearson correlation, and Bonferroni correction was applied to adjust for multiple comparisons.

## 3 RESULTS

**FIGURE 2:**
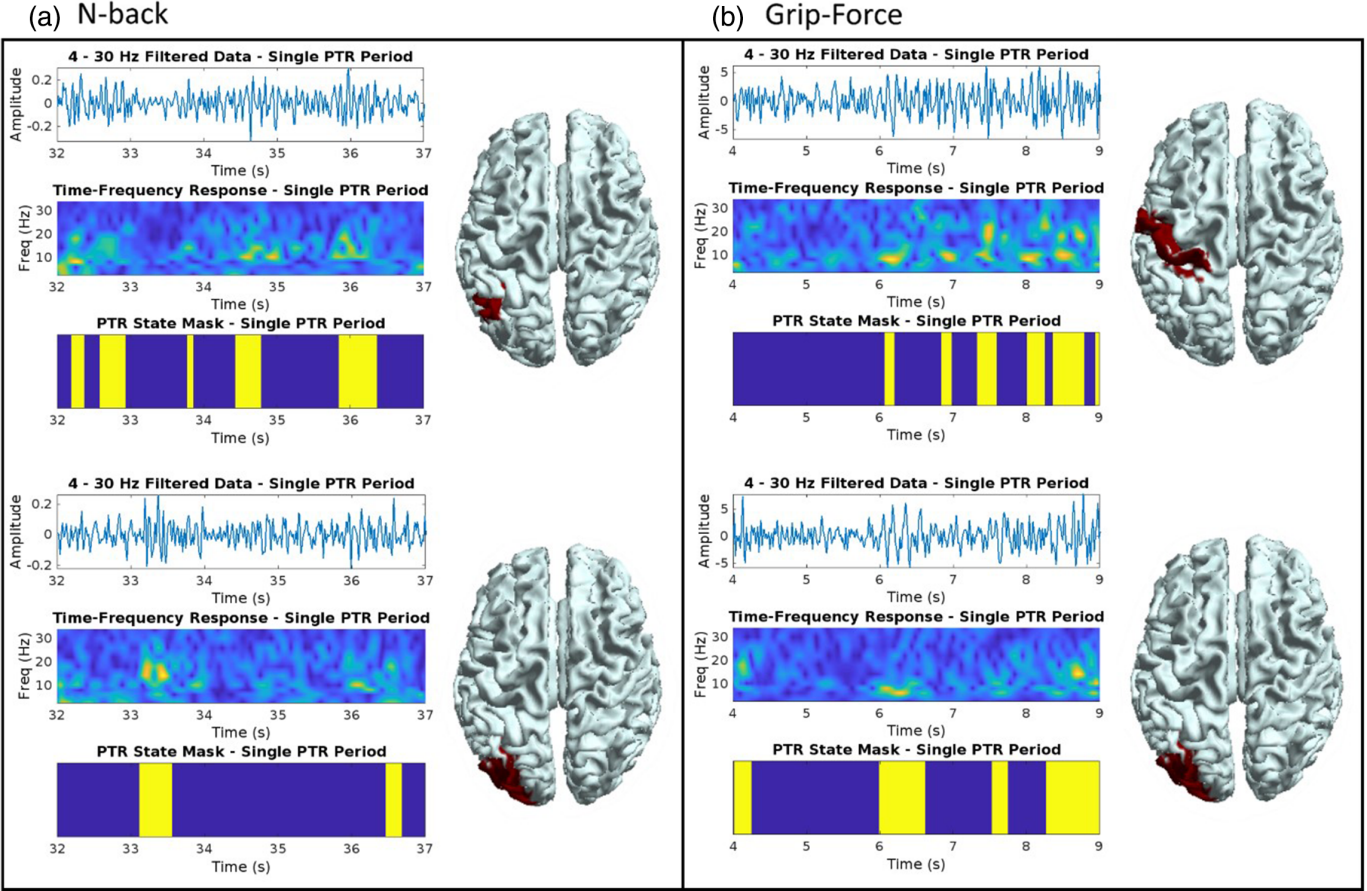
A qualitative comparison of post‐task response (PTR) state events and conventional time–frequency decompositions during example data segments, each starting immediately upon task cessation and lasting 5 s, denoted as the PTR period. The PTR state clearly encompasses transient high amplitude oscillatory events, mostly in the alpha band, but also occurring in the theta and beta bands.

A PTR state was successfully identified over the whole brain for each of the tasks, see Supplementary results (Figure S2). If a higher number of states were chosen the PTR could sometimes be seen in more than one state (Figure S3), seemingly reflecting the contribution of different frequency bands to a burst in a given brain region. Figure 2 provides a visual comparison between PTR state events and single‐trial MEG TFRs during a 10 s data segment during some example PTR periods (immediately post‐task). Each panel shows two AAL regions, as exemplars for each task. In both tasks, the PTR state timing corresponds to transient spectral bursts, that is, short periods of high power. These transient periods of high power seem to appear mostly within the alpha band although are also seen in the theta and beta bands.

**FIGURE 3:**
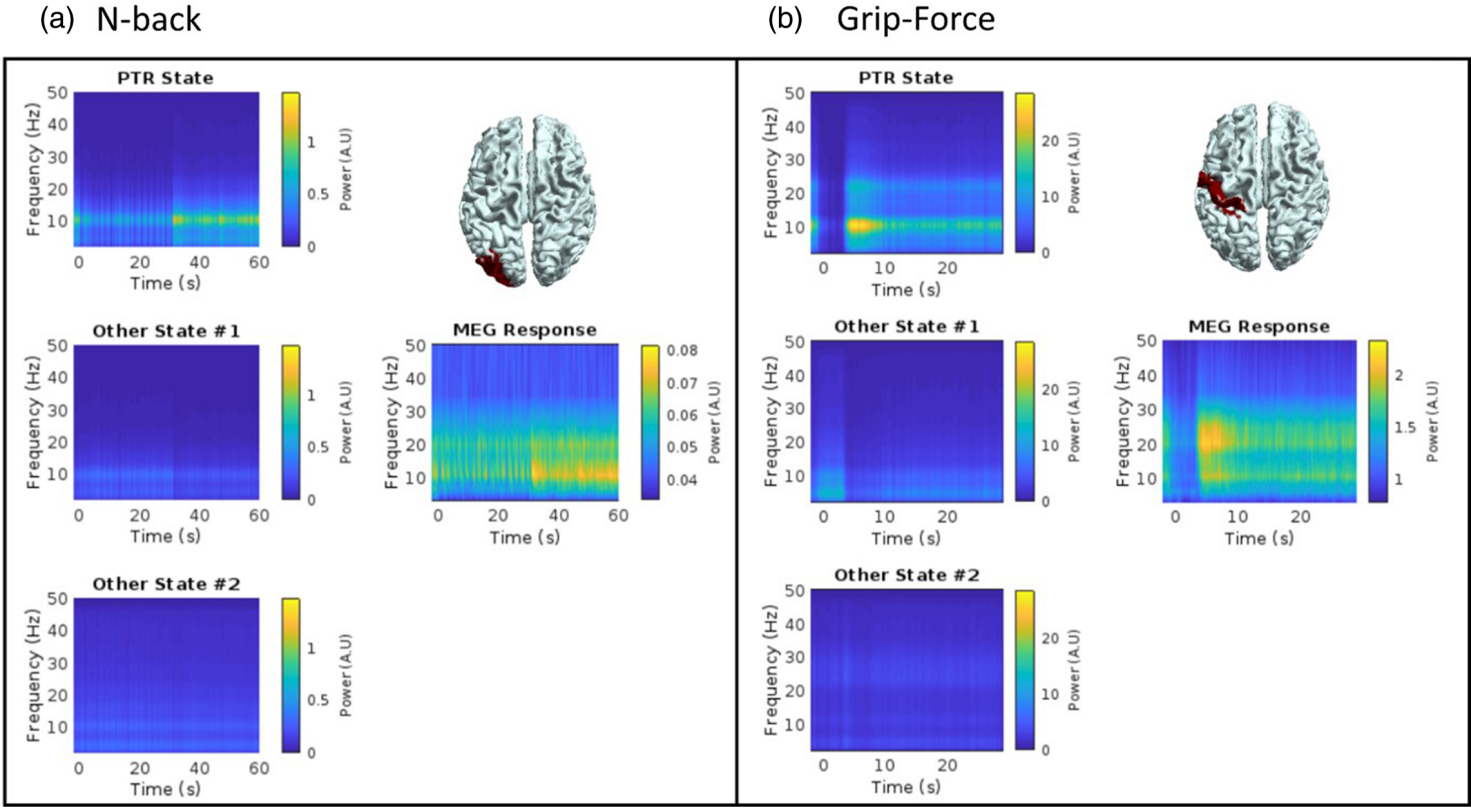
Reconstruction of the time–frequency response using hidden Markov model (HMM) outputs. For each task (panel a: n‐back, panel b: grip‐force), the magnetoencephalography (MEG) response for the automated anatomical labelling (AAL) region pictured is shown on the right side of the panel, for a single condition (highest SNR condition—2‐back for n‐back and 2 s for grip‐force). On the left hand side of each panel are the reconstructed time–frequency responses, using each of the three HMM states.

The PTR state in both tasks captures the during‐task and post‐task induced responses, while the other states seem to reflect lower amplitude events or moments where other activity is *not* happening (satisfying the continuity requirement of HMM state sequences). We demonstrate this below in Figure 3 by comparing the average MEG TFR (right side of each panel, absolute power) to reconstructed TFRs of each of the three states (left side of each panel). Reconstructed TFRs are formed from a matrix multiplication between the PSD curve of the regional (region shown on brain) HMM states, and the average probability evolution of the three states from the same region. From these figures, it is clear that the PTR state represents most of the induced response measured by MEG. However, for both tasks, the alpha band is better represented than the beta band in the PTR state.

**FIGURE 4:**
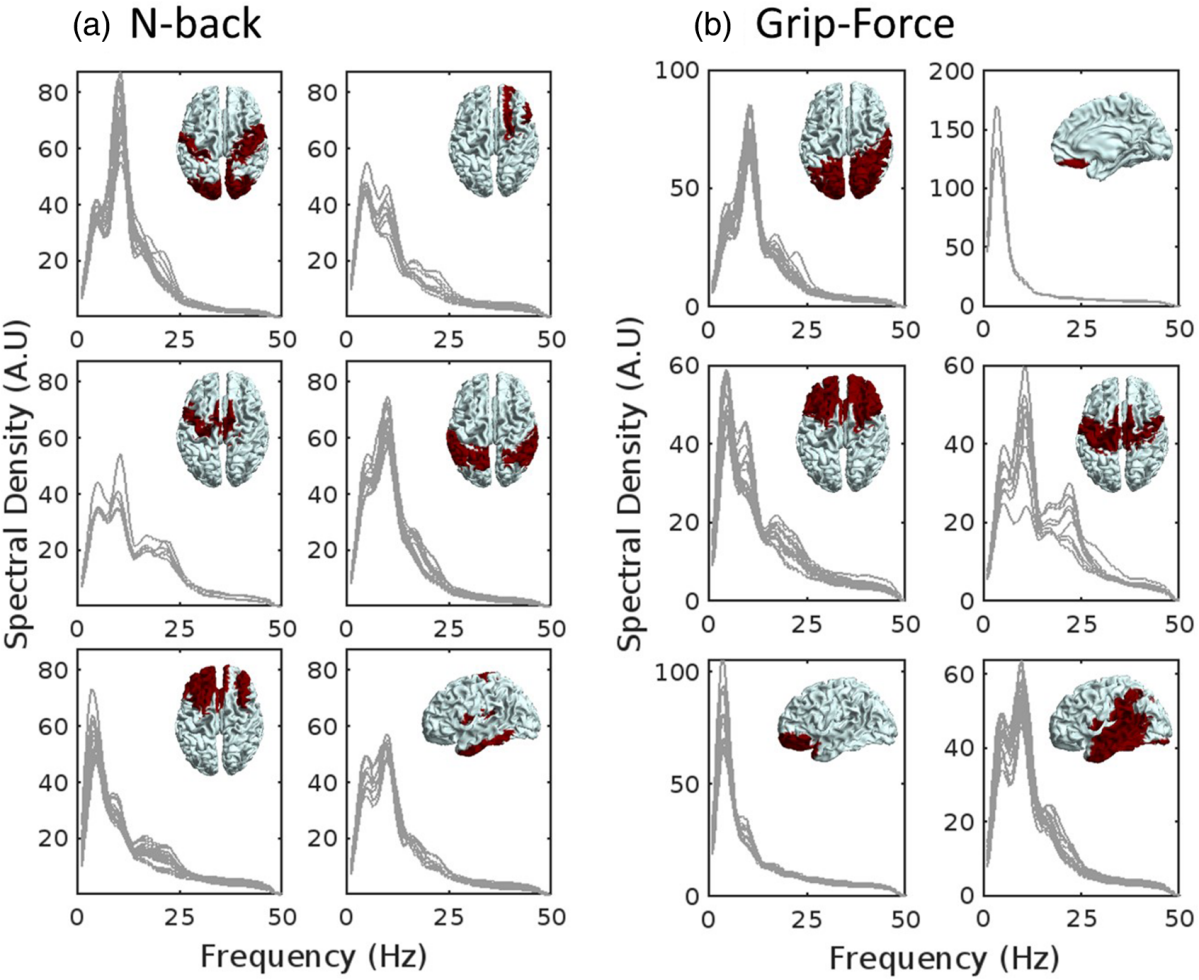
Individual power spectral density (PSD) curves for post‐task response (PTR) states across the cortex, clustered into six groups to aid visualisation.

We have established that the PTR state captures moments of high amplitude spectral activity (Figure 2), which we refer to as *bursts* (Quinn et al., 2018; Seedat et al., 2020), that make up most of the during‐task and post‐task induced response (Figure 3). The spectral content of these bursts is shown in Figure 4, clustered into six groups for each task based on PSD morphology. Clearly, bursts vary in peak frequency based on region; however, the spectral content of bursts within a particular region is relatively narrow.

**FIGURE 5:**
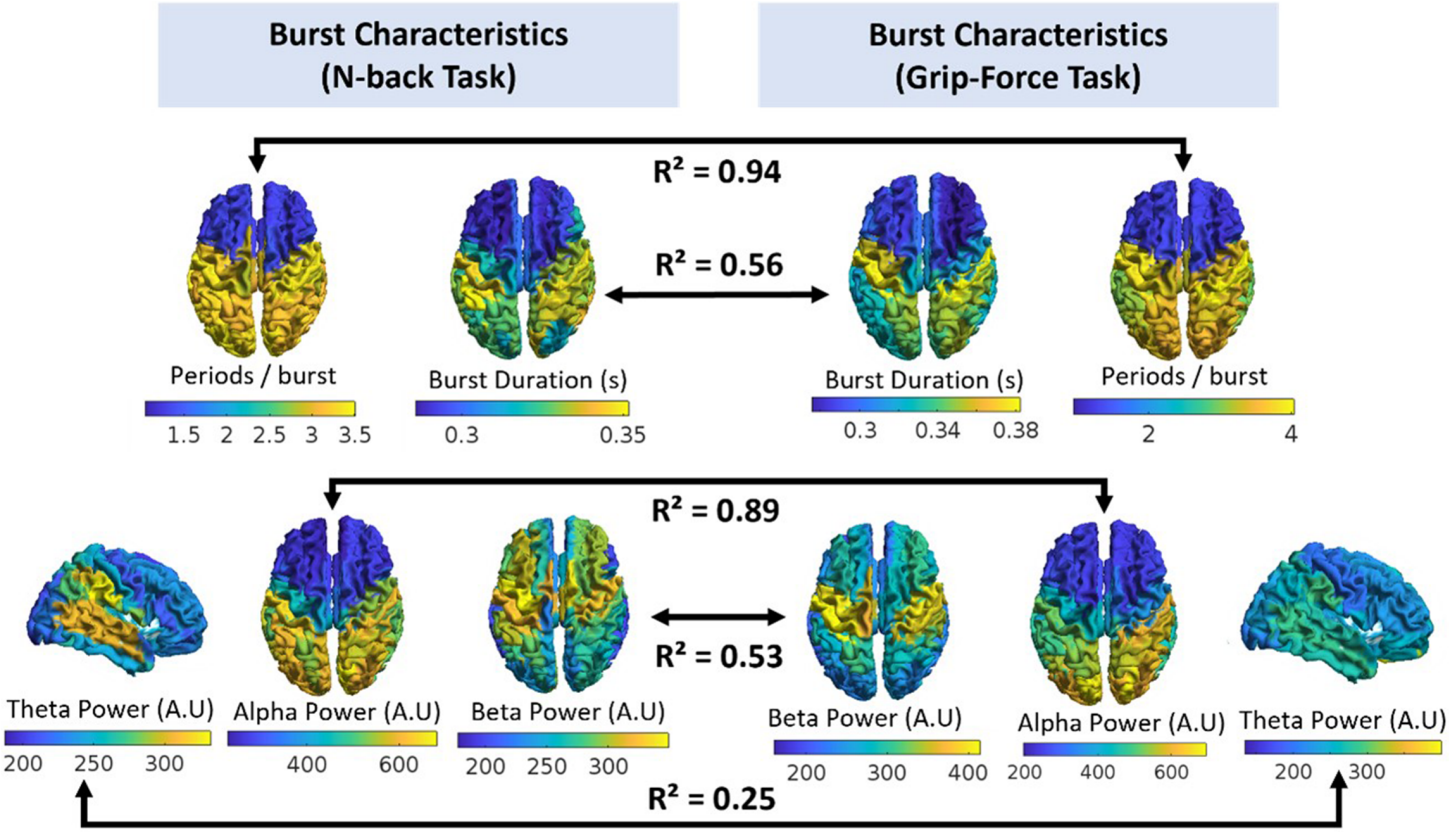
Post‐task response (PTR) state burst characteristics for the n‐back task (left panel) and grip‐force task (right panel). The upper row shows burst durations across the cortex and the number of oscillations per burst (i.e., the burst duration normalised by the peak spectral frequency in the same region). The number of oscillations per burst is almost perfectly correlated between tasks. The lower row shows theta, alpha, and beta activity in the PTR state for the n‐back and grip‐force tasks, distributed over the brain. The two tasks showed high similarity in alpha and beta content across the cortex, whereas theta clearly deviates in relative power between the two tasks.

Burst characteristics of the PTR state are compared in a region‐wise manner between the two tasks, shown in Figure 5. The top row of Figure 5 shows burst durations, as well as oscillatory periods per burst, which equates to the regional burst duration divided by the peak burst frequency in the same region (from the spectra in Figure 4). Burst durations varied similarly across regions when comparing the two tasks with a high level of correlation *R* ^2^ = .56 over all brain regions. The longest durations were seen in the motor strip, followed by posterior regions, while the frontal lobe had notably shorter burst durations in both tasks. After normalising burst durations to peak oscillatory frequency, the two tasks were almost perfectly correlated, with *R* ^2^ = .94, suggesting a fundamental oscillation stability which depends on emitting region. On average over the entire brain, burst durations were found to be 0.31 ± 0.03 s for the n‐back task, and 0.32 ± 0.04 s for the grip‐force task (mean over regions ± standard error over regions).

The lower row in Figure 5 shows spectral power of the PTR state in three canonical frequency bands, for each AAL region. These figures show that for these two different tasks, the PTR state has similar spectral peak locations in each frequency band (theta: temporal and inferior parietal regions, alpha: visual region, beta: motor region), despite variation in relative power across the two tasks. Pearson's correlation results show that alpha and beta power across regions for the PTR state are strongly correlated between tasks. Theta power shows more variation across regions between tasks.

**FIGURE 6:**
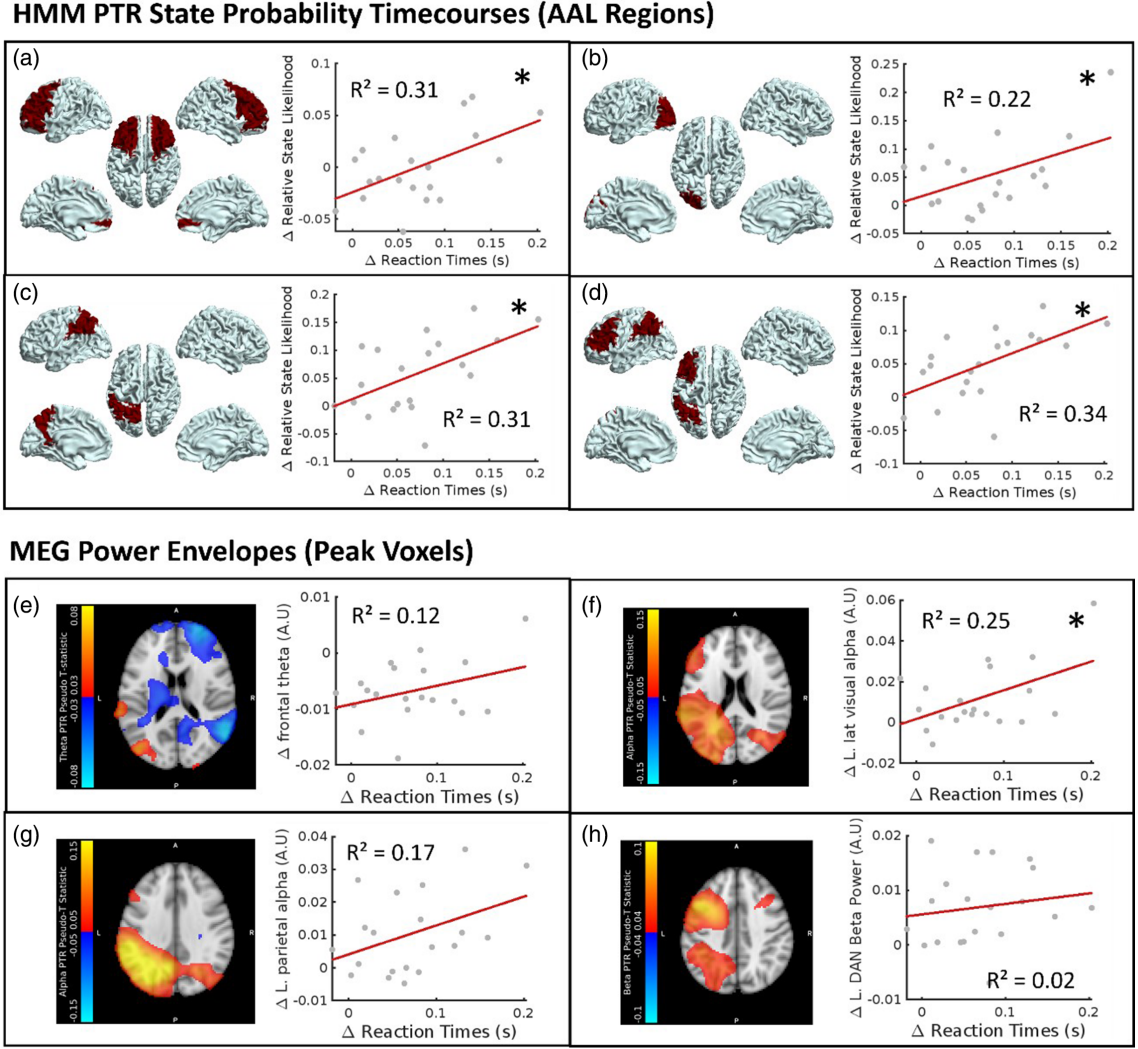
Panels (a–d) show the correlations between post‐task response (PTR) amplitudes in the PTR HMM state, and reaction times, for the n‐back task only. Here we use PTR state probability at the centroids of automated anatomical labelling (AAL) regions. Panels (e–h) show the correlations between magnetoencephalography (MEG) power envelopes from locations of peak pseudo T‐statistic in specific frequency bands and reaction times, adapted with permission from Coleman et al. (2023), * denotes a significant correlation (*p* < .05, Pearson correlation).

As well as providing a useful bursting description of induced brain activity, HMMs also have the ability to isolate activity of interest from other neuronal and physiological signals as well as noise. Here, we show that correlations with behaviour are improved by using HMM state burst probability rather than frequency filtered power envelopes. Figure 6a–d shows the correlations between relative probability of the PTR state during the PTR period (30–37 s) and RTs (averaged over the 0–30 s while the task was occurring). For both of these measures, the difference between 2‐back and 1‐back was taken, as in Coleman et al. (2023). Figure 6e–h shows the correlations between traditional MEG power envelopes and RTs, reproduced with permission (Coleman et al., 2023). Here, we used the HMM PTR state in AAL centroids rather than the MEG power envelopes from peak pseudo‐T statistic locations in anatomical regions (shown in Figure 6e–h). Correlations improved dramatically when using the HMM state rather than the MEG power envelope for all four regions/networks. It is important to note that this correlation is not suggesting that the PTR is predictive of RT, but rather, RTs are predictive of the PTR, as the PTR starts between 2 and 28 s after the RTs it is being related to.

## 4 DISCUSSION

Given the neuroscientific and clinical relevance of the PMBR and beta bursting (Briley et al., 2021; Gaetz et al., 2020; Gascoyne et al., 2021; Little et al., 2019; Seedat et al., 2020; Sherman et al., 2016), it is important that we unify our knowledge of PMBRs with PTRs that occur outside of the motor cortex following higher cognition. This study takes important steps towards this goal, demonstrating that PTRs following visuomotor and working memory tasks are both driven by transient spectral bursts (Figure 2) with similar characteristics (Figure 5). We show that PTRs across both tasks are encompassed by a distinct HMM state, found by running single‐region HMMs and grouping across regions using k‐means clustering (Figures S1 and S2). The PTR state contains both the during‐task and post‐task parts of the response, even when many more states are added to the model (see Figure S3). PTR states across both tasks are primarily in the alpha frequency band (Figure 3), with distinct, relatively narrow band spectral properties in different regions (Figure 4) and similar lifetimes over participants and regions (Figure 5). In addition, we show that the PTR state represents most of the induced oscillatory activity in the MEG response (Figure 3). Finally, we demonstrated the denoising effects of univariate HMMs on MEG data, showing that PTRs in HMM state timecourses have stronger correlations with RTs than the power envelopes (Figure 6).

### 4.1 PTRs across the brain are driven by transient bursts

Recent studies have shown that the PMBR, that is, the post‐task ERS in the beta‐band in the motor cortex, is explained by changes in timing of transient bursts (Brady et al., 2020; Pakenham et al., 2020; Seedat et al., 2020). Interest in bursting is on the rise, with studies attempting to use burst characteristics (timing, burst rate, etc.) as correlates of behaviour or pathology (Briley et al., 2021; Tinkhauser, Pogosyan, Little, et al., 2017; Tinkhauser, Pogosyan, Tan, et al., 2017; Torrecillos et al., 2018). Despite this, current research into bursting outside of the beta‐band and the motor cortex is lacking. Our results in Figure 2 show that the PTR state cluster corresponds to moments of high amplitude activity, in theta, alpha, and beta bands, while our results in Figure 5 show that these events are around 300 ms in duration across tasks, with some variation in duration depending on the emitting region. These burst events make up the induced (time‐locked but not phase‐locked) signals that are observed in trial averaged MEG data (Figure 3, Figure S2). It is important to note that our results show that PTRs are formed of distinct spectral patterns in regions across the brain, *not* a global increase in broadband power, which could be attributed to noise.

In the motor cortex, the region most closely investigated with regard to bursting behaviour, we observe the longest duration of bursts which were 340 ± 20 ms and 390 ± 20 ms (mean ± standard error across subjects) for the n‐back and grip‐force tasks, respectively. Previous studies that have used HMMs to identify beta bursting events have reported burst durations of 300 ms (Seedat et al., 2020) (three state HMM), 200–500 ms (Quinn et al., 2019) (five state HMM), and 300–400 ms (inferred from duration peaks, three state HMM) (Gascoyne et al., 2021). It is difficult to directly compare these values, however the fact that these values all exist in a similar range suggests a common phenomenon was captured by the chosen HMM state in each case. Interestingly, studies using an amplitude thresholding approach for burst detection generally report shorter burst durations, for example, 238 ms (Zich et al., 2023), 213 ms (Pauls et al., 2022), and 94.2 ms (Little et al., 2019). This discrepancy is possibly due to the thresholds used to detect the bursts.

Overall, in the context of previous work, we demonstrate that PTRs across the cortex following working memory and movement are well‐described by a transient bursting model. As with the PMBR case (Pakenham et al., 2020, Seedat et al., 2020), we see that the sustained (after trial‐averaging) induced responses, where a smooth increase (ERS) in band‐specific power is observed, is explained by timing of the transient bursts (Figures 2 and 3 and Figure S2). Prior to this study, burst analysis has been focused on beta‐band responses (Little et al., 2019; Shin et al., 2017), which are usually induced by sensorimotor tasks/stimuli. Our results demonstrate that bursting models may be applicable to all induced responses, despite variation in induced frequency response and emitting regions.

### 4.2 Whole‐brain bursting states can be identified via PTRs

The approach proposed here for mass‐univariate (multiple single region) HMM analysis removed the influence of pre‐defined frequency borders, allowing PTR states in different regions to have distinct spectral properties, as illustrated by Figure 4. This contrasts previous methods which characterised single‐region HMM states by correlating them with frequency filtered power envelopes (Gascoyne et al., 2021; Khawaldeh et al., 2022; Rier et al., 2021; Seedat et al., 2020). Given our results, showing that PTRs across multiple frequency bands are all driven by transient bursting, we suggest that it is highly beneficial for univariate HMM states to be grouped without the constraints of the canonical frequency bands. We propose that k‐means clustering (or similar clustering algorithms) to group univariate HMM states may be useful in identifying induced bursting states across a range of tasks. Based on our results, either the presence of a PTR (given a sufficient rest period) or overall spectral density could be used to identify a state cluster that corresponds to induced bursting. However, this latter marker must be tested with paradigms such as resting state that do not exhibit visible PTRs, to show that the bursting state still holds the highest spectral density if a clear PTR is not present in the data. It is possible that a multilevel HMM (Aarts, 2019) could remove the need for post‐hoc state grouping, allowing for multivariate states to be inferred which allow a certain degree of heterogeneity across “levels” of the model. To our knowledge, multilevel HMMs have not been implemented in neuroimaging analysis.

### 4.3 Burst events across visuomotor and working memory tasks have similar characteristics

To determine whether PTRs following visuomotor and working memory processes are driven by bursts with similar characteristics, burst durations and burst frequency content were compared across tasks. If PTRs in each task were driven by a different type of bursting, we would expect variation in either the global average burst durations (averaged over all regions per participant, then averaged over participants), or the regional burst durations (averaged over all participants per region). The global average burst durations for the PTR state cluster, for the two tasks, were 310 ms for n‐back and 320 ms for grip‐force, within one standard error of each other, which is very similar considering the majority of the participants performing each task were different. Regional variation in burst durations was also very similar tasks, showing high correlation (*R* ^2^ = .56, Figure 5), with the longest (motor strip) and shortest (frontal lobe) burst durations occurring in the same locations. It must be noted that the absolute values may in part be driven by the number of states in the HMM (Seedat et al., 2020). However, as the same number of states was inferred for each region, variation of lifetimes across the cortex should be invariant to this effect, and thus high similarity in cortical variation between tasks (Figure 5) still provides indication that PTRs in both tasks are driven by similar bursts. When burst durations were normalised to the regional peak frequency, the number of oscillations per burst was extremely consistent between tasks (*R* ^2^ = .94, Figure 5), suggesting an intrinsic oscillation stability that depends on emitting region. The number of cycles per burst varies between 1.5 in frontal regions (where low frequency theta activity dominates) and 3.5 in posterior regions (where alpha and beta activity dominate). This result shows that the events we are detecting conform to the concept of “bursts” rather than ongoing oscillations. We performed additional analysis to look for changes in burst duration between time windows, shown in Figure S4.

Spectral content was also used to assess whether PTRs across the two tasks were driven by the same underlying neuronal events. As shown in Figure 5, there is a high correlation between tasks in the alpha and beta power measured across regions. Alpha contributions are consistently highest in posterior and central regions, particularly in parietal and visual regions, while beta contributions are greater in sensorimotor regions. This clearly agrees with the known predominant generators of these two oscillatory bands during rest (Barone & Rossiter, 2021; Clayton et al., 2018; Hindriks et al., 2015; Moran et al., 2019). Interestingly, theta contributions are the greatest in temporal, inferior parietal and frontal regions for both tasks but the correlation of the theta power over the whole brain for the two tasks is weak. We suggest this difference in theta response between the tasks may be due to a larger modulation of theta‐emitting regions (superficial DMN regions) for the n‐back task than the grip‐force task. It must be noted that the in our n‐back dataset we did not see a particularly large frontal theta response, unlike previous studies (Brookes et al., 2011; Missonnier et al., 2006). We believe this may be due to the head positioning in our MEG system meaning the sensors at the front of the system were furthest away, which could be mitigated in future studies using OPM‐MEG (Rhodes et al., 2023). A more prominent frontal theta response would possibly create more divergence between the n‐back and grip‐force tasks.

We hypothesise that alpha power shows the highest similarity across the brain over tasks due to both tasks requiring constant visual processing, thus placing relatively equal load on the visual network across tasks (where alpha is strongest). Beta power shows more variation than alpha power, likely due to higher load on motor regions for the grip‐force task, where motor output had to be kept constant, as opposed to the n‐back task which only required occasional button pressing based on working memory information. In summary, we suggest that variation in the peak frequency of PTRs does not reflect the function of the response, but simply the networks recruited for the task that are being brought to rest. This result supports previous evidence that frequency of induced response depends on network architecture (Elul, 1971; Lopes da Silva et al., 1976; Pfurtscheller et al., 2000; Pfurtscheller & Lopes Da Silva, 1999), as well as evidence that different circuits in the brain have natural frequencies (Rosanova et al., 2009).

Recent studies have reported the existence of harmonic beta activity which arises due to activity in the lower frequency bands (Rodriguez‐Larios & Haegens, 2023; Schaworonkow, 2023). Our results (Figures 2 and 4) show that regions tend to have a distinct spectral profile, meaning beta bursts are not always a simple harmonic of the alpha response (Rodriguez‐Larios & Haegens, 2023, Schaworonkow, 2023). However, we do find that in some regions there are some simultaneous occurrences of alpha and beta bursts (Figure 2b), which can be separated into different states by an HMM with a greater number of allowed states (Figure S3). It is possible that in these cases we are observing harmonic beta activity (Rodriguez‐Larios & Haegens, 2023, Schaworonkow, 2023). This finer delineation of the PTR state(s) warrants further investigation, but we suggest our results also corroborate speculation that alpha and beta should not be distinguished in their function (Griffiths et al., 2019; Hanslmayr et al., 2016).

Together, the results discussed above provide evidence that PTRs following visuomotor and working memory tasks are driven by bursts with similar characteristics, underpinned by a common neural phenomenon. The specific frequency and duration of the bursts appears to be a reflection of the emitting region, which in turn reflects the processes required to complete the task set. We suggest that timing of bursts, reflected by burst rate (how many bursts in a given time window) and burst connectivity (coincidence of bursts, spanning all frequencies, in a given time window), may be more indicative of the underlying function of the response than spectral content. These metrics are shown in Figures S5 and S6.

### 4.4 HMMs isolate functionally relevant features in MEG data

HMMs should, in principle, isolate activity of interest and remove unwanted signals, which may be other neuronal signals or physiological and environmental noise. If PTRs are providing an inhibitory response required to bring recruited networks back to rest, they should relate to behavioural measures of cognitive load such as RTs (Coleman et al., 2023). We hypothesised that by selecting a PTR state using HMMs, correlations of the PTR response with behaviour should increase compared with using the frequency filtered power envelopes. Figure 6 provides strong evidence to support our hypothesis, with the goodness of fit (*R* ^2^ values) increasing dramatically in left parietal (*R* ^2^ = .17–.31) and frontal (*R* ^2^ = .12–0.31) regions when moving from frequency filtered power envelopes to HMM PTR state probability. In addition, a strong relationship was found in the left DAN (*R* ^2^ = .34) which had an *R* ^2^ = .02 when using oscillatory power in the beta band. Here, we clearly show that between‐subject variation in RTs, reflecting perceived task difficulty, is predictive of PTR amplitudes occurring after the task has ended. This supports the various models of PTRs relating to inhibitory control (Chen et al., 1998; Engel & Fries, 2010; Gaetz et al., 2010; Mullinger et al., 2017; Neuper & Pfurtscheller, 2001; Pfurtscheller et al., 2005; Salmelin et al., 1995; Solis‐Escalante et al., 2012; Zhang et al., 2008), expanded in Coleman et al. (2023) to include pan‐spectral PTRs following working memory. It should be noted that the correlation between RT and left lateral visual PTRs decreased slightly in the HMM states compared to the power envelope (*R* ^2^ = .25–0.22; *p* = .024 to *p* = .038). This may be because the effective location used when averaging AAL segments in the HMM timecourse was far away from the point of peak activity. From Coleman et al. (2023), this seems likely, as the peak activity overlapped with the temporal lobe. Given the structural (rather than functional) basis of the AAL segmentation, this was hard to replicate without including the full temporal lobe, a region which was not studied in Coleman et al. (2023). Overall, this finding supports the hypothesis that spectral bursts, that become more probable post‐task, serve an inhibitory function to bring active networks to resting levels of activity, and therefore relate to behavioural markers of cognitive load.

## 5 CONCLUSION

We have shown that PTRs following movement and working memory are both driven by transient burst events. These bursts, encompassed in a single HMM state for each region, drive induced changes in both the during‐task and post‐task time windows. The PTR state cluster showed remarkable similarities across tasks. Burst durations across participants fall within a standard error of each other between tasks and show highly correlated spatial patterns for both tasks, with the longest durations in the motor strip, and shortest in the prefrontal cortex. The PTR state showed very similar alpha and beta variation across the brain between tasks, with greater difference in the theta band activity that is likely driven by differences in the network recruitment. Overall, these results suggest that differences in frequency content of PTRs reflects the emitting regions, and thus the recruited network architecture, rather than variation in PTR function across tasks. Together our results suggest a similar bursting phenomenon is being measured in both the visuomotor and working memory task, driving PTRs in each case. For the n‐back task, correlations between PTRs and RTs were improved by using HMM PTR state timecourses rather than frequency filtered power envelopes from the same region, demonstrating the ability of HMMs to isolate signals of interest, and corroborating the hypotheses relating PTRs to inhibitory control of network activity.

## CONFLICT OF INTEREST STATEMENT

The authors declare no conflicts of interest.

## Supporting information


**DATA S1:** Supporting Information.

## Data Availability

The data that support the findings of this study are available on request from the corresponding author. The data are not publicly available due to privacy or ethical restrictions.
