# Supplementary material for: Post‐task responses following working memory and movement are driven by transient spectral bursts with similar characteristics
Source: Hum Brain Mapp. 2024 May 10;45(7):e26700. doi: 10.1002/hbm.26700 (PMC11082833; doi:10.1002/hbm.26700)
Supplement: Supplementary file 1 — DATA S1: Supporting Information. [file HBM-45-e26700-s001.docx]

**Supplementary Information**

**Supplementary Methods**

*K-means Clustering Simulation*

Our approach to state grouping used k-means clustering in a high dimensionality space where each timepoint corresponded to a separate axis, such that a single point represents the entire time evolution of an HMM state, and therefore temporally similar states are more readily clustered. We tested the reliability of clustering states with this method using a set of simulated timecourses to ensure that the clusters were meaningful, given the high dimensionality of the clustering. A set of three cluster centroids were simulated using random binary timecourses, each comprising 1000 timepoints (leading to 1000 axes in the clustering space). These were replicated 50 times each, to create 3 perfect clusters, where cluster members were identical to their respective cluster centroid. Next, a varying amount of noise was added to each cluster, by setting a switching probability (%), which dictated the chance of each timepoint switching value (0 to 1, or 1 to 0). Given that the timecourses were binary, a switching probability of 50% corresponds to complete randomisation of the timecourse. Switching probability values tested were 10%, 20%, 30% and 40%. Following the addition of noise, the k-means clustering algorithm was applied to the set of simulated timecourses, and the cluster assignment values were imaged next to the matrix of timecourses to show the clustering accuracy.

*10 State HMM*

Using a 3 state HMM, the PTR state describes the induced responses during the task/stimulus as well as the PTR itself. In the case of the well-studied motor cortex response to movement, the PTR state contains both the MRBD (ERD) and the PMBR (ERS). It is possible that this is purely due to using a low number of states, i.e., the autocovariance of bursting during the stimulus/task and during the PTR may be similar but different enough to be separated if the model is provided enough freedom. We tested this by performing a 10 state univariate HMM on representative regions from each task dataset and plotting the probability timecourses for each state.

*Burst durations in different time windows*

Burst duration results presented in the main paper were all based on the entire scan period. Here, we investigate whether burst durations within the PTR state cluster change based on task timings, e.g. during the task and post-task time windows. To do this, the binary state timecourses were segmented by the task time window (n-back: 0.5 – 29.5 s, grip-force: 4 s starting 1 s before grip start) the post-task time window (n-back: 30 – 37 s, grip-force: 4 s starting 2 s after grip cessation), and the rest window (n-back: 40 – 60 s, grip-force: 4 s starting 21 s following grip cessation) separately. It is worth noting that time-windows were hard-masked, i.e., bursts that took place on the boundary between time widows were truncated prior to the calculation of state lifetimes - this was deemed acceptable given that state lifetimes were much shorter than the chosen time windows.

*Burst Rate*

We argued in the main paper that, given similarity in bursts between different tasks, burst timing may be more indicative of underlying brain function than the intrinsic properties of individual bursts. Burst rate, i.e., number of bursts in a given time window, is one metric that describes timing of bursts. Burst rate was calculated during the task, post-task and rest time windows, and investigated in two ways: 1) averaged over all regions per participant, and 2) averaged over all participants per region, to provide a complete picture of how burst rate varied between time windows.

*Burst Coincidence*

Another metric related to burst timing is burst coincidence, i.e., similarity in burst timing across the cortex. Coincidence of burst across the brain, regardless of frequency band, may provide important information about underlying network dynamics. We used the Jaccard index, as previously used in (Seedat et al., 2020), as a measure of functional connectivity of the PTR state across the brain in different time windows. We took the change in Jaccard index when moving from the task to post-task time windows for each dataset to view the effects of PTRs on network activity in the two tasks.

**Supplementary Results**

Figure S1 shows the effect of noise on the accuracy of k-means clustering, when grouping a set of simulated HMM state timecourses. Clusters were assigned perfectly at switching probability values of 10%, 20% and 30%. Clustering accuracy broke down at 40% switching probability (80% noise), although most of the timecourses in each simulated cluster were still assigned correctly. This shows that the k-means algorithm is working as expected, grouping states based on temporal similarity even when a substantial amount of noise is added. Highly connected HMM states were grouped across the brain using k-means clustering, with the aim of grouping states that captured PTRs. The results of this clustering are shown in Figure S2. The left hand side of each panel shows the cluster centroid timecourses, which are equivalent to the average of all state timecourses in the cluster. From these, the PTR state can clearly be identified as cluster #1 in the n-back task (Fig. S2, panel A), and cluster #3 in the grip-force task (Fig. S2, panel B), as these clusters reflect the MEG responses reported in (Coleman et al., 2023) and (Pakenham et al., 2020), respectively. It should be noted that for the n-back task, there is no visible PTR for the 0-back and 1-back conditions in the cluster centroid #1 as the response for these conditions was much weaker in many regions (Coleman et al., 2023), likely due to very low working memory load of these two conditions compared to the 2-back condition. We also show the Euclidian distance of each state from the cluster centroid as colourmaps over the brain (right hand side of each panel). Here, the lower values (cooler colours) are favourable as they represent closer temporal matching between the state and the cluster centroid, i.e., a highly connected cluster. In both tasks, the PTR state is the most highly connected state cluster (i.e. time courses over the different regions match the most closely).


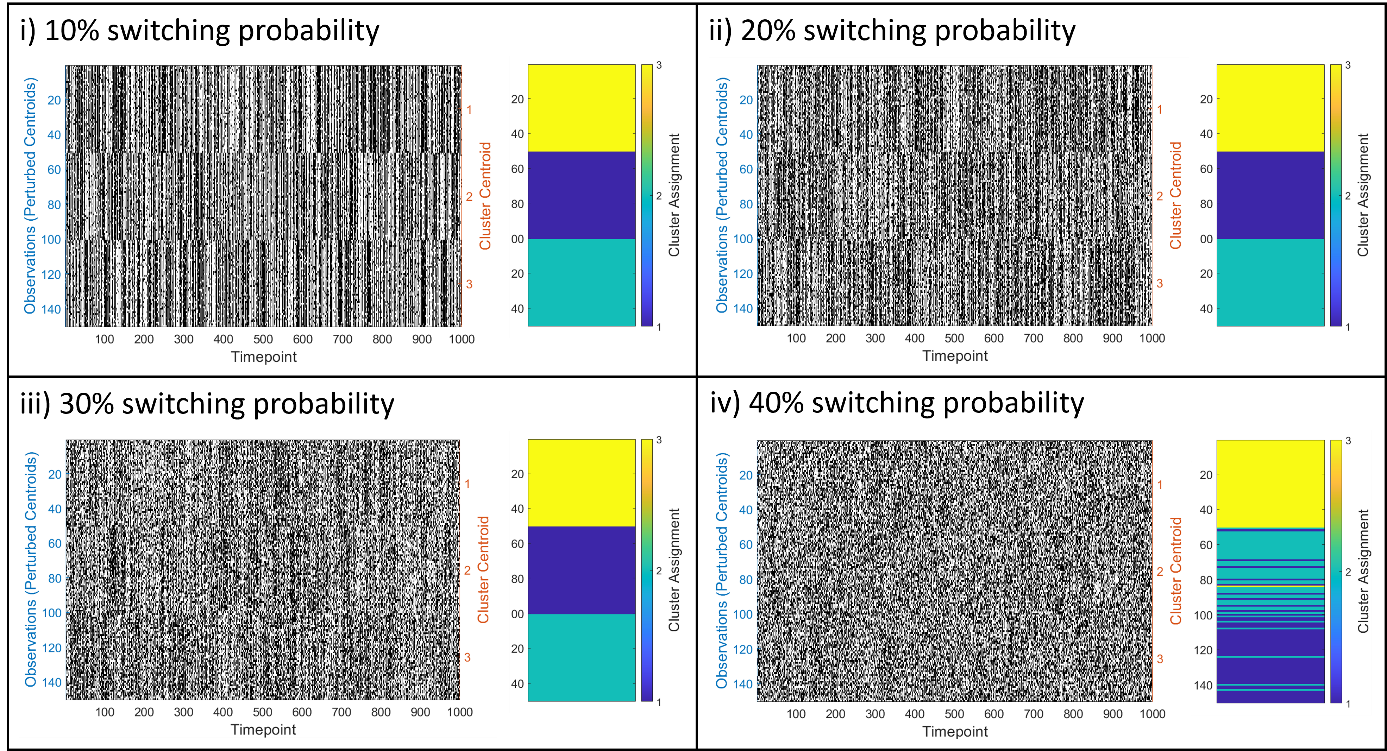


Figure S1: A simulation demonstrating the ability of the k-means clustering algorithm to group HMM states based on temporal similarity. The matrix on the left of each panel contains three simulated clusters of binary HMM state timecourses, with an amount of noise denoted by the switching probability (% chance for each timepoint to switch value, independently). The result of applying k-means clustering to each matrix is shown by the vector on the right of each panel. For 10%, 20% and 30% switching probability, every single timecourse was assigned correctly. Clustering accuracy broke down at 40%, which is close to complete randomisation of the timecourses (which occurs at 50%). These results show that the k-means clustering approach to grouping HMM state timecourses is robust to a substantial amount of noise.


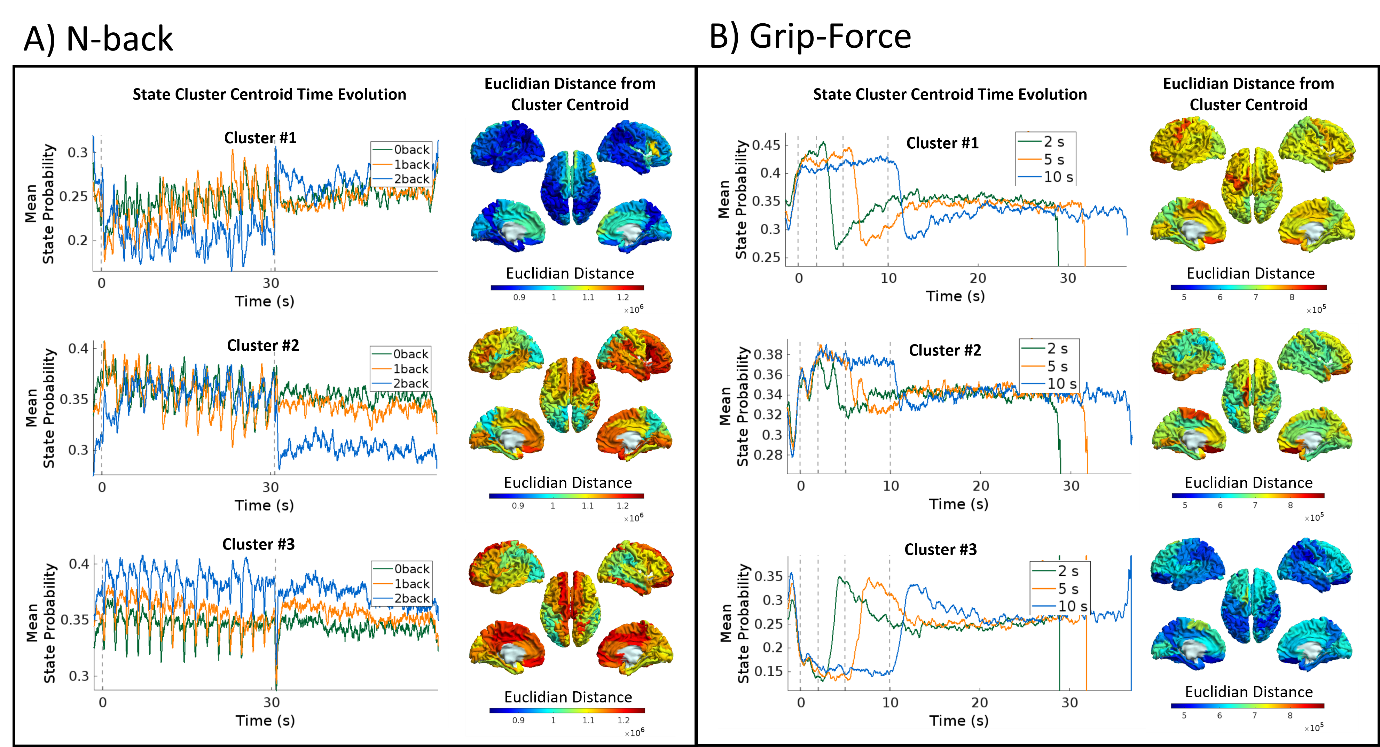


Figure S2: The three state clusters, found by grouping single-region HMM states over regions using k-means clustering, based on temporal similarity of the state timecourses. For each task (A: N-back, B: Grip-Force) the cluster centroid is shown, equivalent to the average of all state probability timecourses in the cluster. The PTR state cluster can easily be identified as cluster #1 for the n-back task and cluster #3 for the grip-force task. For each cluster, the Euclidian distance of each state from the cluster centroid is shown as a colourmap over the brain, representing the quality of the clustering (right hand side of each panel). In these images, low Euclidian distance (cool colours) represents high temporal similarity between the state and the cluster centroid. The PTR state cluster has the lowest Euclidian distances (highest cluster quality) for both tasks. Note a different colour bar is used for the Euclidean distances for the two tasks. For individual representative region timecourses and comparison with 10 state HMM see Figure S3.

Figure S3 shows the results of performing a 10 state HMM on representative regions for each task. For each state, the probability timecourse is shown, i.e., the binary state timecourse averaged over all trials/blocks for each task condition. For both tasks, the during-task response is never separated from the PTR. Given the results discussed in the main paper, this suggests that phases of the induced response (during-task, post-task) occur due to changes in burst probability, not changes in spectral content of bursts, as this would result in a state with an isolated PTR (i.e., no during-task modulation). For the grip-force task, the 10 state HMM was able to separate burst of differing frequency content (panel Bii), which could be useful in studying genuine vs harmonic beta bursts (Schaworonkow, 2023, Rodriguez-Larios and Haegens, 2023).


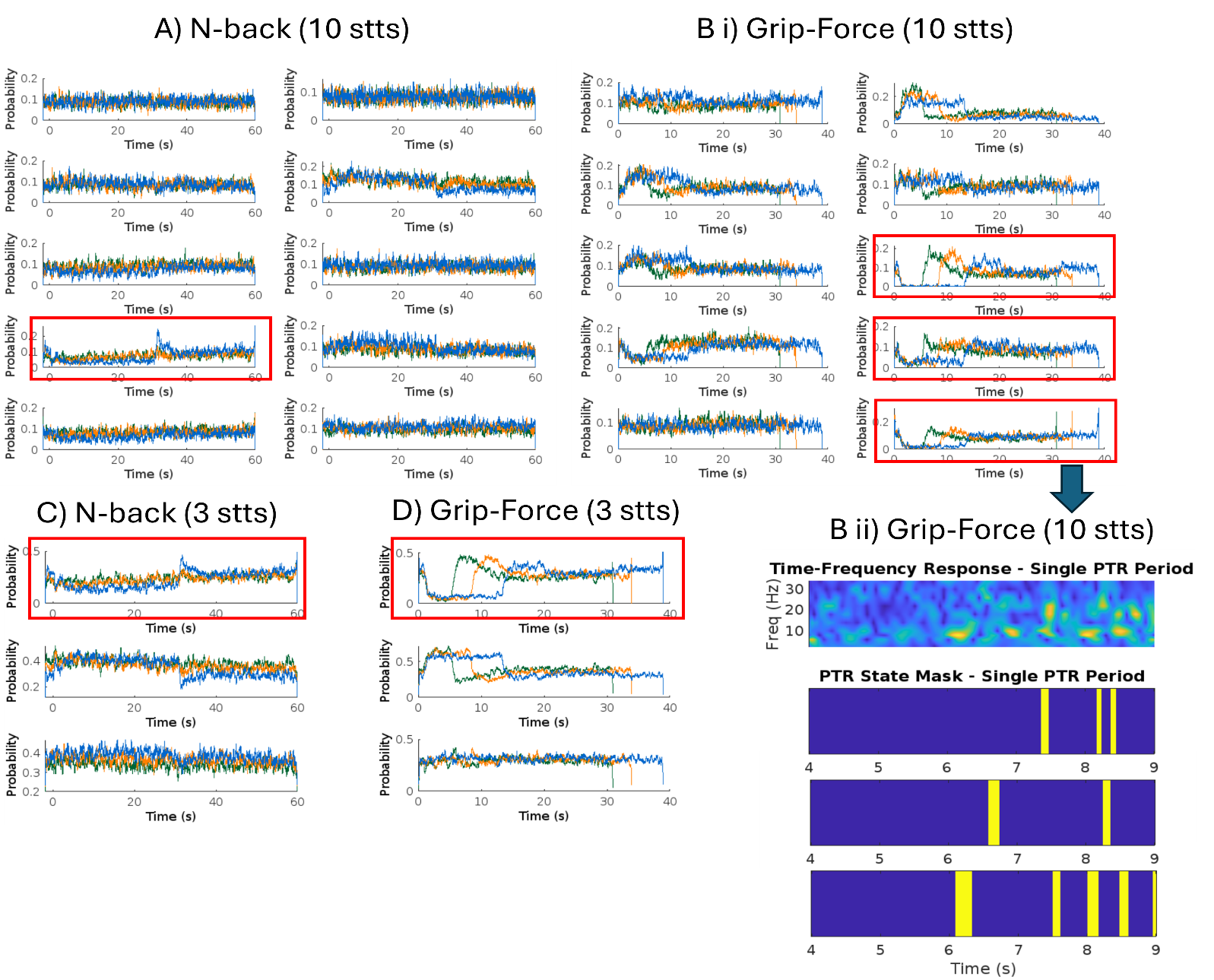


*Figure S3: HMM run with 10 states for the n-back task (panel A) and the grip-force task (panel B) for two representative regions. Trial-averaged state probability timecourses are shown for each state. Panel Bii shows that the 10 state HMM separated bursts of different frequencies for the grip-force task, resulting in three PTR states in panel Bi. The 3 state HMM timecourses are shown in panels C and D for the n-back and grip-force tasks, respectively. These 3 state timecourses contributed to the cluster centroid timecourses shown in Figure S2.*

Figure S4 shows the results of measuring PTR state lifetimes (burst durations) in different time windows, averaged over all regions per participant (Fig. S4 A-B) and averaged over all participants per region (Fig. S4 C-D). Burst durations modulate significantly (p < 0.05, rm-ANOVA) between time windows for both tasks, with the task window containing the shortest durations. This may be a reflection of the change in spatial distribution of activity between time-windows, which can be seen in changes in burst rate (see Fig. S5). For both tasks, bursts are more probable in frontal regions during the task/stimulus, and these have the shortest burst durations (Fig. 4), whereas bursts in motor and parietal regions become more likely during the PTR, and these have longer burst durations (Fig. 4).


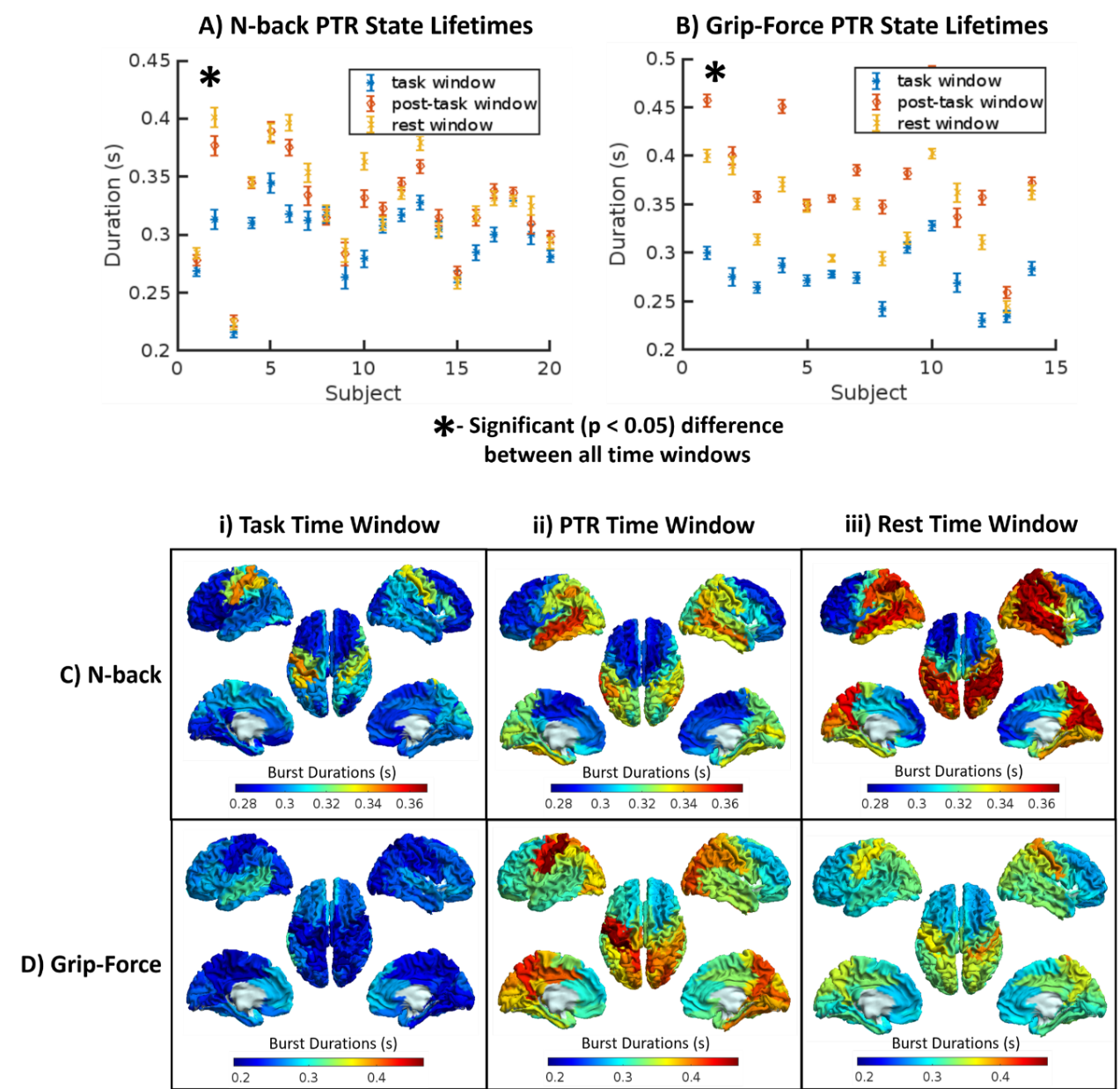


*Figure S4: State lifetimes, equivalent to burst durations, for the PTR state cluster, averaged over all regions for each participant (A, B), and averaged over all participants for each region (C, D) for the n-back (A, C) and grip-force (B, D) tasks. A significant modulation of the state lifetimes (* p < 0.05, rm-ANOVA) was found over participants between time windows for both tasks.*

Burst rate is shown below in Figure S5 in different time windows for each task. In line with our speculation in the main paper, this metric shows greater divergence between tasks, possibly reflecting differences in task-specific processing. It is also worth noting that spatial differences we observe here, i.e., high state occurrence in frontal regions during the task time window, and greater motor/parietal/visual occurrence frequencies during the PTR and rest windows, may explain the modulations in average lifetime we observe in Figure S4.


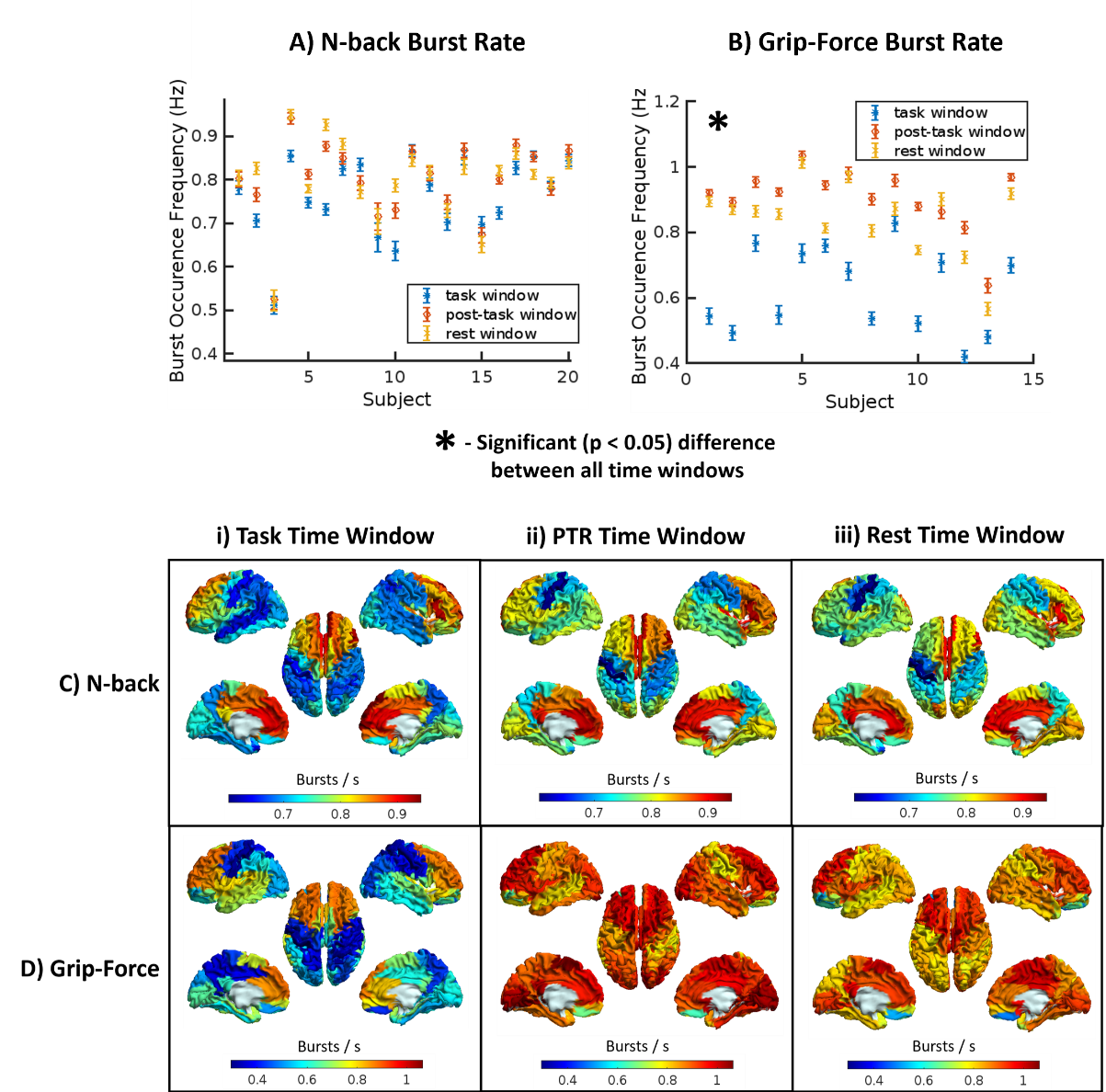


*Figure S5: Burst rate for the PTR state cluster, averaged over all regions for each participant (panels A and B), and averaged over all participants for each region (panels C and D) for the n-back (A, C) and grip-force (B, D) tasks. A significant modulation (* p < 0.05, rm-ANOVA) was found over participants between time windows, for the grip-force task only.*

Figure S6 shows the changes in Jaccard index values (connectivity) when moving from the task time window (n-back: 0.5 – 29.5 s, grip-force: 4 s starting 1 s before grip start) to the post-task time window (n-back: 30 – 37 s, grip-force: 4 s starting 2 s after grip cessation). For the n-back task, superficial DMN regions (frontal, temporal, inferior parietal) see the greatest change in connectivity during the PTR. For the grip-force task, bilateral visual, parietal, and left motor regions see the greatest change in connectivity. These results reflect networks known to be modulated by each task. Alpha/beta oscillations are thought to relate to top-down inhibition in neuronal circuits (Pfurtscheller et al., 1996, Chen et al., 1998, Jensen et al., 2002, Halgren et al., 2019, Klimesch et al., 2007), therefore increased synchronicity during the PTR may reflect top-down inhibition through intra-cortical networks. Future work is required to further elucidate the role of the PTR in re-establishing resting state network activity.


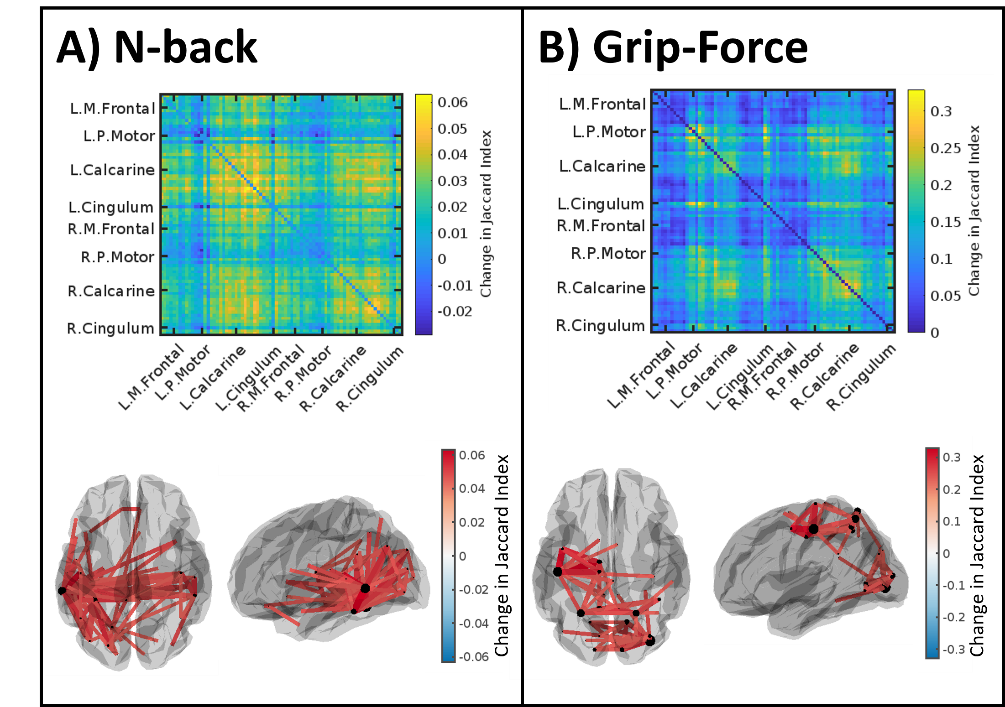


*Figure S6: Functional connectivity, as measured with Jaccard index, representing changes in coincident bursting* *when moving from the task time window (n-back: 0.5 – 29.5 s, grip-force: 4 s starting 1 s before grip start) to the post-task time window (n-back: 30 – 37 s, grip-force: 4 s starting 2 s after grip cessation) for the two tasks (n-back (A) and grip-force (B)). The top 30% of values in the connectivity matrices are displayed in the glass brain plots.*

**References**

CHEN, R., YASEEN, Z., COHEN, L. G. & HALLETT, M. 1998. Time course of corticospinal excitability in reaction time and self-paced movements. *Ann Neurol,* 44**,** 317-25.

COLEMAN, S. C., SEEDAT, Z. A., WHITTAKER, A. C., LENARTOWICZ, A. & MULLINGER, K. J. 2023. Beyond the Beta Rebound: Post-Task Responses in Oscillatory Activity follow Cessation of Working Memory Processes. *Neuroimage,* 265**,** 119801.

HALGREN, M., ULBERT, I., BASTUJI, H., FABÓ, D., ERŐSS, L., REY, M., DEVINSKY, O., DOYLE, W. K., MAK-MCCULLY, R. & HALGREN, E. 2019. The generation and propagation of the human alpha rhythm. *Proceedings of the National Academy of Sciences,* 116**,** 23772-23782.

JENSEN, O., GELFAND, J., KOUNIOS, J. & LISMAN, J. E. 2002. Oscillations in the alpha band (9-12 Hz) increase with memory load during retention in a short-term memory task. *Cereb Cortex,* 12**,** 877-82.

KLIMESCH, W., SAUSENG, P. & HANSLMAYR, S. 2007. EEG alpha oscillations: the inhibition-timing hypothesis. *Brain Res Rev,* 53**,** 63-88.

PAKENHAM, D. O., QUINN, A. J., FRY, A., FRANCIS, S. T., WOOLRICH, M. W., BROOKES, M. J. & MULLINGER, K. J. 2020. Post-stimulus beta responses are modulated by task duration. *Neuroimage,* 206**,** 116288.

PFURTSCHELLER, G., STANCAK, A., JR. & NEUPER, C. 1996. Post-movement beta synchronization. A correlate of an idling motor area? *Electroencephalogr Clin Neurophysiol,* 98**,** 281-93.

RODRIGUEZ-LARIOS, J. & HAEGENS, S. 2023. Genuine beta bursts in human working memory: controlling for the influence of lower-frequency rhythms. *bioRxiv***,** 2023.05. 26.542448.

SCHAWORONKOW, N. 2023. Overcoming harmonic hurdles: genuine beta-band rhythms vs. contributions of alpha-band waveform shape.

SEEDAT, Z. A., QUINN, A. J., VIDAURRE, D., LIUZZI, L., GASCOYNE, L. E., HUNT, B. A. E., O'NEILL, G. C., PAKENHAM, D. O., MULLINGER, K. J., MORRIS, P. G., WOOLRICH, M. W. & BROOKES, M. J. 2020. The role of transient spectral 'bursts' in functional connectivity: A magnetoencephalography study. *Neuroimage,* 209**,** 116537.
